# Robust Recombinant Expression of Human Placental Ribonuclease Inhibitor in Insect Cells

**DOI:** 10.3390/biom12020273

**Published:** 2022-02-08

**Authors:** Beáta Flachner, Krisztina Dobi, Anett Benedek, Sándor Cseh, Zsolt Lőrincz, István Hajdú

**Affiliations:** TargetEx Ltd., Madách I. u. 31/2., H-2120 Dunakeszi, Hungary; flachner@targetex.com (B.F.); dobi@targetex.com (K.D.); benedek@targetex.com (A.B.); cseh@targetex.com (S.C.)

**Keywords:** ribonuclease inhibitor, quantitative PCR, baculovirus-insect expression system, high-level soluble expression

## Abstract

Ribonuclease inhibitors (RIs) are an indispensable biotechnological tool for the detection and manipulation of RNA. Nowadays, due to the outbreak of COVID-19, highly sensitive detection of RNA has become more important than ever. Although the recombinant expression of RNase inhibitors is possible in *E. coli*, the robust expression is complicated by maintaining the redox potential and solubility by various expression tags. In the present paper we describe the expression of RI in baculovirus-infected *High Five* cells in large scale utilizing a modified transfer vector combining the beneficial properties of Profinity Exact Tag and pONE system. The recombinant RI is expressed at a high level in a fusion form, which is readily cleaved during on-column chromatography. A subsequent anion exchange chromatography was used as a polishing step to yield 12 mg native RI per liter of culture. RI expressed in insect cells shows higher thermal stability than the commercially available RI products (mainly produced in *E. coli*) based on temperature-dependent RNase inhibition studies. The endotoxin-free RI variant may also be applied in future therapeutics as a safe additive to increase mRNA stability in mRNA-based vaccines.

## 1. Introduction

Ribonuclease inhibitors are a 50 kDa cysteine and leucine-rich repeat protein found in the cytosol of cells in most mammalian species. RI is a potent inhibitor of the ribonuclease A (RNase A) superfamily, inhibiting RNase A activity with an extremely low Ki value of 4 × 10^−14^ M at a molar ratio of 1:1.

RNase A is one of the most prominent causes of contamination both in genetic engineering and in diagnostic polymerase chain reaction (PCR)-based approaches. As RNase A is a very stable and robust enzyme, its total inhibition is required in most cases. The recombinant expression of mammalian RIs represents a very challenging task: its amino acid composition, especially the 28–32 cysteines (the number varies between different species), which must be kept in a reduced form to maintain enzymatic activity. Furthermore, its fold, characterized by alternating units of an α-helix and β-strand forming a striking horseshoe shape [1], also requires a proper folding machinery. Previous data indicate that although RI could be expressed in *E. coli* [2,3,4,5] and *S. cerevisiae* [6] and *D. melanogaster* [7], the robust production of RI requires special expression constructs and conditions.

The oxidation state of RI is a critical factor. All cysteines must be in a reduced form for activity and in the free form the cysteines cooperatively form disulfide bridges [8]. After initial oxidation of a small number of cysteine residues, a conformational change occurs, resulting in the cooperative formation of 15 disulfide bonds that leads to the inactivation of the porcine RI. Disseminating the role of individual cysteine residues, it was shown that Cys328 and Cys329 of human RI is mainly responsible for the initiation of oxidation and their mutation to alanine significantly decreases the sensitivity of RI towards oxidation [9].

RI was originally purified from placenta [10], while the first recombinant RI (porcine) in an active form was expressed in *S. cerevisiae* [6], at a low yield (0.2 mg recombinant protein/g wet cells). The first successful attempt to express RI in *E. coli* yielded 0.25 mg RI/L culture in 1989 [11]. The solubility of RI is very low and its expression in *E. coli* often leads to inclusion bodies. Later modification of the expression procedure led to higher yields: 15 mg/L culture [3] for porcine RI. Addition of the reducing agent dithiotreitol (DTT), low production temperature and co-expression of the chaperonin GroELS resulted in high level production using the conditions with the EnBase^®^ technology [5,12]. Another promising attempt was to use N-terminal tags to increase the solubility of RI. Three protein tags: maltose binding protein (MBP), N-utilization substance A (NusA) and translation initiation factor 2 domain I (IF2) have improved the solubility of murine RI, enhancing the recombinant protein yield to 24–34 mg/L culture [4]. Expression of RI in insect cells was also accomplished in stably transfected *D. melanogaster* cells to yield 1.4 mg/L recombinant human RI [7]. Although several methods were published for the expression of RI, these processes require special conditions or a multi-step purification streamline with addition cleavage of tags, which make them hardly reproducible in common laboratories.

Currently with the continuous spreading of COVID-19 and the discovery of new variants [13,14], the need for diagnostic PCR consumables rapidly increases and therefore sufficient quantity of RI is highly demanded. To minimize the occurrence of false test results, the stability of all components of the diagnostic tests, including RI, should be maximized. The *E. coli* derived “hard to produce” recombinant proteins often have small conformational inaccuracies mainly at the termini, which make these proteins less stable than their native counterparts. In our work, we focused on the expression of RI in the Baculovirus Expression Vector System (BEVS) system where the chance of correct folding and producing more stable proteins is significantly higher than in *E. coli*.

## 2. Materials and Methods

### 2.1. Materials

All chemicals used in the highest commercially available purity. For DNA manipulation, enzymes were purchased from NEB, the synthetic gene was produced at GeneArt (Thermo Fisher, Waltham, MA, USA). Media for recombinant protein expression was purchased from Thermo Fisher (Waltham, MA, USA). Chromatography cartridges and media were purchased from Bio-Rad (Hercules, CA, USA) and Cytiva (Washington, DC, USA). Cell lines *Sf9* and *High Five* were purchased from Thermo Fisher (Waltham, MA, USA), and *Sf-9* ET from Kerafast (Boston, MA, USA).

### 2.2. Transfection, Virus Propagation and Protein Expression

Recombinant RI was expressed in baculovirus/insect cell expression system [15], which is based on the co-transfection of insect cells with linearized *Autographa californica* multiple-capsid nuclear polyhedrosis virus (AcMNPV) baculovirus DNA and a transfer plasmid carrying the gene of interest, for the construction of baculovirus vectors. *Sf9* cells were co-transfected with the previously constructed expression contained the gene for the fused ribonuclease inhibitor gene in pOPal30 vector and flashBAC GOLD (Oxford Expression Technologies) in a 35 mm Petri dish. To obtain p1 and p2 virus stocks, virus infections were conducted over a 7-day period in T75 and T175 flasks, respectively. To obtain a larger amount of viruses for high scale expression, propagation was transferred into shaking flasks. A 180 mL volume of *Sf9* cell culture was grown in 500 mL flasks to 2 × 10^6^/mL density with at least 80% viability. Cells were infected using a multiplicity of infection (MOI) [16] of 0.1 and grown for a further 4 days at 110 rpm agitation at 27 degrees. To quantify the titer of the newly produced virus, stock *Sf9* Easy Titer Cell Line (*Sf-9* ET) was used as suggested [17].

*High Five* cells were infected with an MOI of 0.05 of the virus stock at a cell density of 2 × 10^6^/mL using 2500 mL in each 5 mL shaked flask. Flasks were agitated at 90 rpm at 27 °C. After 1 h, 1% (*v*/*v*) of 50 mg/mL bovine serum albumin (BSA) stock solution was added to the cultures and the flask was incubated for further 95 h.

### 2.3. Purification of RI

At the end of production, the whole fermentation culture was sonicated using Bandelin SONOPULS ultrasonic sonicator equipped with VS70T probe for 5 min at 70% intensity with 60% cycle parameter. The homogenized culture was filtered through an 0.45 µm cellulose acetate filter. The filtered culture was applied to Profinity Exact column, washed with 100 mM sodium-phosphate buffer (pH 7.2) and eluted with 100 mM sodium-phosphate, 100 mM sodium-fluoride (pH 7.2) buffer. For efficient cleavage of the tag, the flow was stopped for 40 min before the collection of the eluted fractions.

The eluted fractions containing RI were collected and subjected to a Q Sepharose Fast Flow anion exchange column in the original elution buffer. After sample load, the column was washed with 40 mM Hepes (pH 7.2) and 40 mM Hepes, 150 mM sodium-chloride (pH 7.2) until equilibrium. The concentrated RI was eluted with 40 mM Hepes, 1 M sodium-chloride (pH 7.2).

The eluted protein was subsequently gel filtrated through a Sephadex G-25 Medium column in 40 mM Hepes, 100 mM sodium-chloride (pH 7.5) buffer. After elution, 16 mM DTT and equal volume of glycerol was added to result a 20 mM Hepes, 50 mM sodium chloride 8 mM DTT, 50% glycerol (pH 7.2) storage buffer for ribonuclease inhibitor. For extended storage, the protein solution was stored at −20 °C.

### 2.4. Temperature Dependent RNase Activity Measurements

Activity measurements are based on the degradation of total RNA by bovine RNase A. Total RNA was isolated from 3T3 mouse cell line using a Monarch Total RNA preparation kit. RI (0.5 mg) was incubated at the indicated temperatures for 30 min before adding to the mix (RNase A 5 pg, RNA 2 µg, buffer: 100 mM Tris, 1.2 mM EDTA, 0.1 mg/mL BSA, 8 mM DTT (pH 7.5). Samples were then incubated at 37 °C for 30 min (to allow RNase to degrade RNA, if active), 6× Loading Dye was added to stop the reaction, and then loaded on 1.2% agarose e-gel. Degradation of RNA was analyzed after imaging with Bio-Rad Gel Doc EZ Imager (Hercules, CA, USA).

### 2.5. DSF Measurements

Temperature-induced unfolding of the proteins was examined by Thermal Shift Assay [18] in a Bio-Rad CFX 96 qPCR equipment based on the differential binding of Sypro Orange fluorescent dye to native and unfolded proteins. For the measurements, all ribonuclease inhibitors were diluted to 0.05 mg/mL concentration in 50 mM sodium-phosphate, 50 mM sodium-chloride (pH 7.5) containing a 1000-fold dilution of Sypro Orange. For the studies in the presence of RNase, bovine RNase A was added in an equimolar ratio with RI. Unfolding was followed between 30–90 °C using a 1 °C/min gradient. Thermal unfolding was defined as the negative peak of the first derivative of the fluorescence curve measured using the FRET channel of the qPCR equipment.

## 3. Results and Discussion

### 3.1. Design of Expression Plasmid

The vector design for the recombinant expression of RI in insect cells is based on two previous vector systems: the Profinity Exact system [19], which utilizes an immobilized, engineered, fluoride triggered subtilisin [20] that both recognizes and avidly binds to the small N-terminal co-expressed affinity tag in a protein fusion; and pONE [21] in which the baculovirus transfer vector (pONE30A) is optimized to yield high protein quantity as a fusion protein. An insect cell codon optimized version of the fusion section of pPal8 was introduced to pONE30A at NdeI/NotI sites to create a new baculovirus transfer vector named pOPal30 compatible with the original Profinity Exact system. A codon-optimized version of the human placental ribonuclease gene was subcloned into pOPal30 using BamHI and NotI sites. The sequence of ribonuclease gene contained two mutations previously identified as the main cause of oxidation resistance: Cys328Ala and Cys329Ala [9]. The sequence of the expression plasmid was verified by sequencing and used for co-transfection of Sf9 with flashBAC GOLD genetically optimized *A. californica* nucleopolyhedrovirus DNA [22].

### 3.2. Expression of RI

The expression of RI in *High Five* cells was originally established in 500 mL shaker cultures using 125 mL medium using different starting cell density in the range of 1.5–3 × 10^6^/mL. Our results showed that the highest amount of recombinant protein could be obtained at 2 × 10^6^/mL cell density. In the 500 mL flasks we optimized the medium level to reach the highest possible amount at 180 mL based on the works of Rieffel et al. [23]. The effect of MOI on the expression level was investigated in the range of 0.01–0.05 and we concluded that 0.05 was the optimal ratio.

The further stage of upscaling of the production was the use of 5 L shaking flasks with 1800–2500 mL medium. The highest amount of RI could be harvested after 96 h, however, as cells partially lysed, proteases degraded the recombinant protein. To confine this proteolytic effect, bovine serum albumin (BSA) was used as a bait for proteases at 0.5 mg/mL starting concentration. The time of addition for BSA was also optimized and we found that its addition after only 1 h after infection worked optimally.

### 3.3. Purification

Recombinant RI was harvested after 96 h, with the protein found both in the supernatant of cells and within cells. We attempted to purify the protein from both sources after centrifugation and work with both fractions individually. From the precipitate the recombinant protein could be retrieved with low efficiency, hence we omitted the centrifugation step. We used a gentle ultrasound sonication step for cell disruption from the whole cell culture including media and centrifuged it only after the disruption step.

The cleared lysed culture was applied to Profinity Exact columns in halogen-free extraction medium. After applying the whole sample to the column, one column volume elution buffer was applied, and the flow was stopped for 40 min at room temperature. After the incubation time, the cleaved protein was eluted with the fluoride-containing elution buffer.

**Figure 1:**
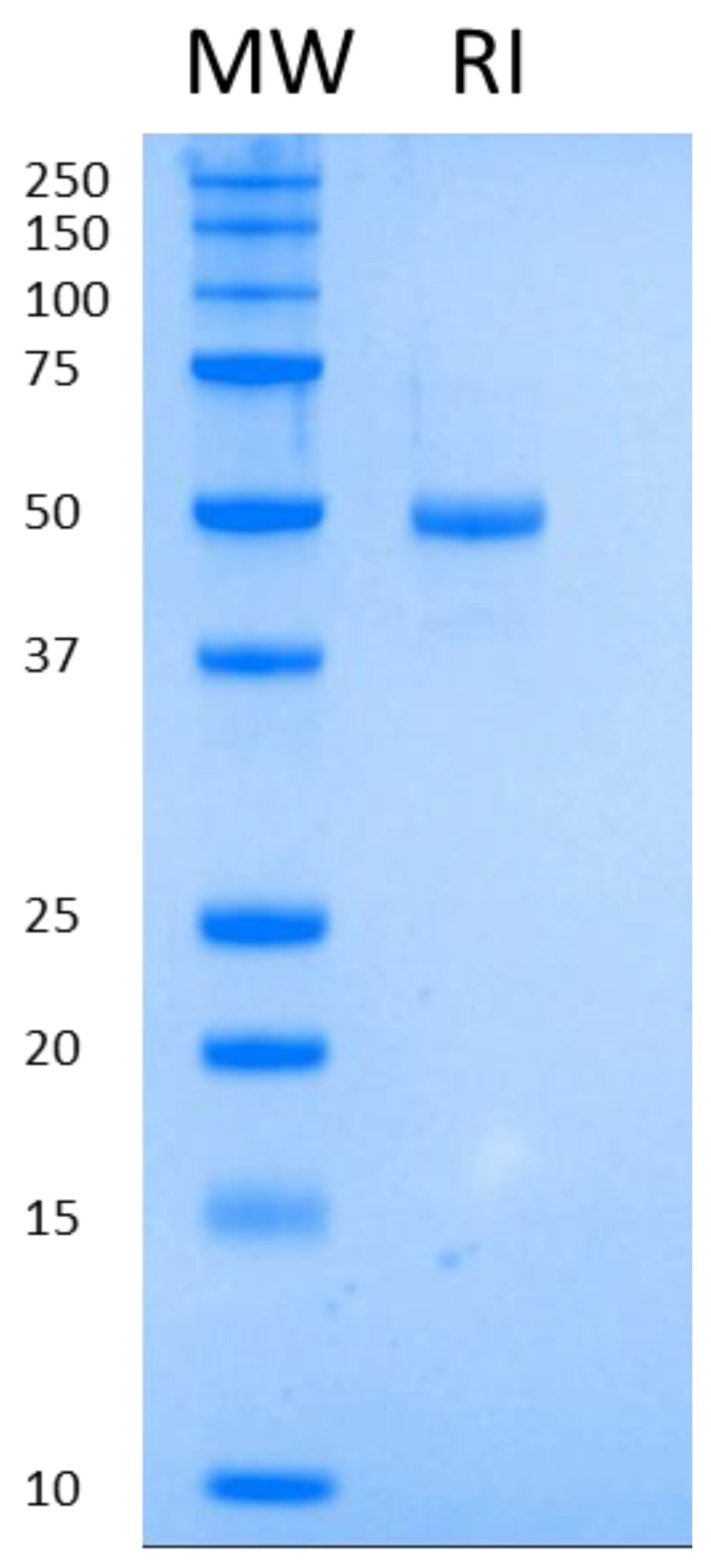
Purified RI run on SDS-PAGE.

The second step of purification is an anion exchange (Q Sepharose Fast Flow), which serves as a three-headed tool: elimination of protein impurities; elimination of host cell DNA and a concentration step. We managed to elute the protein of interest at 5–10 mg/mL concentration in DNA-free form. As a final step, buffer exchange was executed on a Sephadex G-25 column. The final protein was at least 95% pure as visualized on SDS-PAGE (Figure 1).

To quantify the volumetric production of RI, we repeated the expression/purification process several times. The yield of the individual expression of sixteen cycles is shown in Supplementary Table S1. We determined the number of cells, the volumetric yield and yield related to number of infected cells for each cycle. The volumetric yield is 12.4 ± 3.7 mg/L culture, while it corresponds to 3.6 ± 1.1 mg/10^9^ insect cells. The yield is well comparable to that shown in the case of porcine RI in *E. coli* [3].

### 3.4. Heat Stability of RI

**Figure 2:**
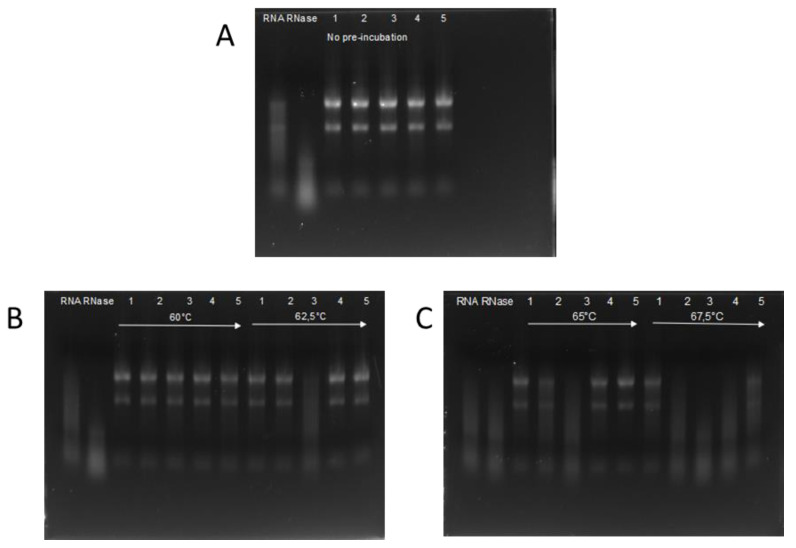
Remaining activity of RI after 30 min incubation at elevated temperatures. In each block, pre-heat temperatures are indicated. RNA is run on an e-gel after incubation with RNase A and the pre-heated RI. In all measurements, lane 1 denotes ‘insect RI’; lane 2 ‘RI I’, lane 3 ‘RI II’; lane 4 ‘RI III’; and lane 5 ‘RI IV’. RNA denotes the experiments without adding either RNase nor RI, and lane RNase is with added RNase, but no RI. **Panel A**: Remaining activity of RIs with no pre-incubation. **Panel B**: Remaining activity of RIs with pre-incubation at 60 °C and 62.5 °C on the left and right side respectively **Panel C**: Remaining activity of RIs with pre-incubation at 65 °C and 67.5 °C on the left and right side respectively.

The activity of RI was compared to four RIs available from commercial sources. RNase activity was investigated using the RI variants previously incubated at 60; 62.5; 65; and 67.5 °C. As a negative control a ‘no RI’ and for positive control not-incubated RI was used. Figure 2 shows that RI expressed in insect cells was able to inhibit RNase completely until 65 °C and partially at 67.5 °C. ‘RI III’ and ‘RI IV’ are fully active until 65 °C, but completely inactive after preincubation at 67.5 °C. ‘RI I’ is fully inhibiting RNase activity until 62.5 °C, and partially at 65 °C. ‘RI II’ is only effective until 60 °C preincubation and is completely inactivated after 62.5 °C treatment.

The results are summarized in Table 1.

**Figure 3:**
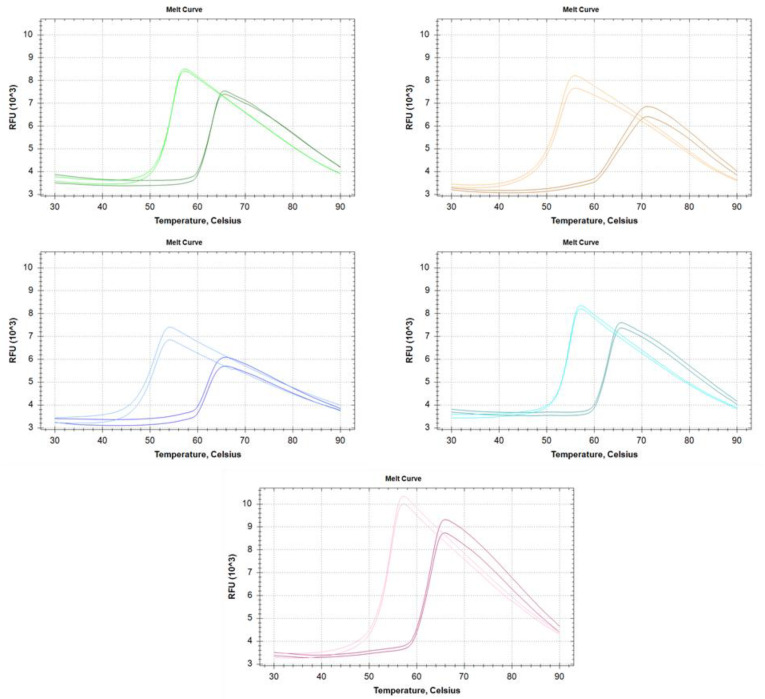
Melting profile of each ribonuclease inhibitor alone and in the presence of excess RNase A. Gree-Insect RI; Orange-‘RI I’; Blue-‘RI II’; Turquoise-‘RI III’; Pink-‘RI IV’. On every panel, the lighter colored curves correspond to free RI, while the darker for the RI-RNase A complex.

The thermostability of the RI variants was investigated using Thermal Shift Assay [18] in the absence and presence of RNase A (Figure 3).

The melting temperature of each enzyme is shown in Table 2. Interestingly, the melting temperatures of the enzymes do not directly correlate with the results derived from heat activation measurements. Without RNase A, the melting temperatures vary in the range of 50.5–54.6 °C, and the binding of RNase A increases the melting temperature by 8.6–12.4 °C to reach 62.9–64.1 °C. The profiles of the melting curves indicate simple two-state unfolding processes, however ’RI I’ shows a much lesser cooperative unfolding thermogram in complex with RNase A.

Ribonuclease inhibitor expressed in insect cells shows the highest thermostability based on the residual activity after incubation at elevated temperatures in the set of RIs examined. This behavior is of high interest for biotechnological purposes as insect-expressed RI can be used at higher reaction temperatures and for longer storage time of intact samples. The biological property behind the elevated thermotolerance can lie in the stability of the protein itself or the ribonuclease-RNase complex, as it is well known that RNase significantly stabilizes RI as it is demonstrated by the increase of melting temperature of the protein [24]. We have shown that although the RI variants show smaller differences in the melting profiles; there is no direct relationship between the biophysical thermostability and the residual activity after heat treatment of the variants. It is also worth mentioning that several variants show elevated heat resistance even above the melting temperature of the RNase-RI complex.

A new perspective could rise for insect derived RIs with the high success of mRNA vaccines [25]. A major drawback of the current mRNA-based vaccines is their storage at ultra-low temperature due to their low stability [26]. It was suggested that addition of RI to the mRNA-lipid nanoparticles could help preventing the damage of mRNA structure [27,28]. The addition of RI at local administration of naked self-replicating mRNA significantly improved its repeatability and efficacy [29]. We should emphasize that RI expressed in *High Five* cells is per se endotoxin-free, which is a high priority in vaccine development [30,31].

## 4. Conclusions

To our knowledge, recombinant human placenta ribonuclease inhibitor was successfully produced in baculovirus/insect cell system for the first time. The volumetric quantity is comparable to that from *E. coli*, but the process does not require special modifications of the basic strategy of the expression system. The thermal stability of RI produced in *High Five* exceeds its *E. coli* counterparts making it a strong candidate to be used in biotechnological applications in the future.

## Figures and Tables

**Table 1:** Remaining activity of different ribonuclease inhibitors after incubation at the marked temperatures.

	60 °C	62.5 °C	65 °C	67.5 °C
Insect RI	+	+	+	partial
‘RI I’	+	+	partial	−
‘RI II’	+	−	−	−
‘RI III’	+	+	+	−
‘RI IV’	+	+	+	−

**Table 2:** Melting temperatures of the different ribonuclease inhibitors in the absence and presence of RNase A.

-	Melting Temperature (°C)	
	- RNase A	+ RNase A
Insect RI	54.1	63.2
‘RI I’	52.2	63.3
‘RI II’	50.5	62.9
‘RI III’	54.5	64.1
‘RI IV’	54.6	63.2

## Data Availability

The data used to support the findings of this study are available from the corresponding author upon request.

## Supplementary Materials

The following supporting information can be downloaded at: https://www.mdpi.com/article/10.3390/biom12020273/s1, Table S1: Ribonuclease inhibitor yield in a set of experiments.
