# Supplementary material for: Robust Recombinant Expression of Human Placental Ribonuclease Inhibitor in Insect Cells
Source: Biomolecules. 2022 Feb 8;12(2):273. doi: 10.3390/biom12020273 (PMC8961516; doi:10.3390/biom12020273)
Supplement: Supplementary file 1 [file biomolecules-12-00273-s001.zip › biomolecules-1551244-supplementary.pdf]

Supplementary Table S1. Ribonuclease inhibitor yield in a set of experiments

Exp. No.	Cell number	Yield (mg RI/10^9 cells)	Yield (mg RI/liter culture)
1	3.1E+10	3.3	10.3
2	4.9E+10	4.6	17.9
3	3.2E+10	3.1	8.7
4	3.1E+10	5.4	16.6
5	3.9E+10	4.6	15.8
6	3.4E+10	3.3	11.5
7	5.0E+10	3.3	13.5
8	3.5E+10	5.5	18.9
9	4.5E+10	4.2	14.8
10	3.7E+10	3.1	11.6
11	4.8E+10	1.7	8.3
12	2.8E+10	3.7	10.6
13	3.1E+10	2.1	6.5
14	3.2E+10	4.4	14.2
15	3.9E+10	3.3	10.7
16	4.4E+10	2.2	7.9
Average	3.8E+10	3.6	12.4
St. Dev.	7.4E+09	1.1	3.7
CV%	19.5	30.3	30.2
